# Nurses’ Transitions Into and Out of the Home Health Sector

**DOI:** 10.1177/10775587261422386

**Published:** 2026-02-18

**Authors:** Zoe Samson, Lauren J. Hunt, Laura M. Wagner, Janette Dill, Ulrike Muench

**Affiliations:** 1 University of California San Francisco, USA; 2 MaineHealth Center for Nursing Research and Quality Outcomes, Portland, ME, USA; 3 University of California San Francisco School of Medicine, USA; 4 Healthforce Center at University of California San Francisco, USA; 5 University of Minnesota Twin Cities, Minneapolis, USA

**Keywords:** nursing, workforce, home health

## Abstract

Demand for home health is growing. Registered and licensed practical/vocational nurses (RNs and LPN/LVNs) are integral to the provision of home health services, but a limited body of evidence suggests that the workforce is unstable and possibly shrinking. We assessed RNs’ and LPN/LVNs’ transitions into and out of the home health sector using data from the Current Population Survey monthly files from January 2003 to June 2023. Specifically, we compared turnover from home health versus the hospital sector and assessed entry into home health from other health care and non–health care sectors. Turnover was consistently and significantly higher in the home health sector compared with the hospital sector. The predicted probability of nurses entering home health from any other sector hovered around or below 1%. High turnover combined with low home health entry raises concerns about home health nursing services nationwide.

## Introduction

A variety of home-based services exist in the United States to address a spectrum of care needs (Harrison et al., 2020). Registered nurses (RNs) and licensed practical or vocational nurses (LPNs/LVNs) play a central role in delivering skilled clinical home health services—a Medicare benefit available following hospitalization or in response to other acute health needs (Centers for Medicare and Medicaid Services, 2020). Other forms of home-based care, which do not typically involve RN and LPN/LVN services, include home-based primary care delivered primarily by physicians and nurse practitioners (Leff, 2024; Yao et al., 2021), and daily care supports provided by informal- often family-caregivers or by home health/care aides paid privately or through Medicaid-reimbursed home and community-based services (Medicaid and CHIP Payment and Access Commission, 2025). Home health needs in the United States are expected to rise dramatically due to large numbers of seniors aging into Medicare (Centers for Medicare and Medicaid Services, 2023) and rebalancing of long-term care services out of facilities and into homes (Bernacet et al., 2021). This has led to projections that home health care services will be among the top 10 fastest-growing industries between 2022 and 2032, with a 20.2% increase in demand for employment over that time span (Colato & Ice, 2023). Calls to build robust home-based clinical care also became more pronounced following the COVID-19 pandemic, when the low capacity of facility-based health care failed to address patients’ needs (Ritchie & Leff, 2022). By measuring the movement of RNs and LPN/LVNs into and out of the home health sector at the national level, we aimed to better understand the capacity of this workforce to meet home-based clinical care needs.

Nurses are an integral part of the home health clinical workforce. However, staffing and turnover raise concerns that the demand for home health services is exceeding the supply of nurses available or willing to provide this type of care. Recent estimates show that approximately 30% of RNs and LPN/LVNs leave their jobs annually and that home health agencies maintain vacancy rates at approximately 25% (Hospital and Healthcare Compensation Service, 2022). Home health workforce shortages may already be contributing to unmet care needs, as suggested by growth in post-acute home health referral rejections (CarePort, 2023).

Concerns about the stability of the home health nursing workforce exist in a broader landscape of shortage concerns across the entire nursing profession. Cycles of nurse turnover were already long-standing (Seo & Spetz, 2013; Spetz, 2004; Spetz & Given, 2003) before the COVID-19 pandemic. The pandemic drew attention to the challenges of nursing and health care workforces amid amplified burnout and turnover (Galanis et al., 2021; Martin et al., 2023), particularly in settings with pre-existing adverse work environments (Aiken, Sloane, et al., 2023). A 2022 national-level survey showed that over one quarter of U.S. RN and LPNs/LVNs were considering leaving the profession entirely over the subsequent 5 years (Smiley et al., 2023). However, most recent nursing turnover analyses are concentrated in acute hospital settings (Aiken, Lasater, et al., 2023; Woodward & Willgerodt, 2022), and we lack evidence focused on home health nurse turnover, both before and following the onset of the COVID-19 pandemic.

Despite distinct challenges including unpredictable and hazardous home environments, long commutes, and limited clinical and administrative support (Agbonifo et al., 2017; Bien et al., 2020), home health nurses once ranked their work environments more positively than hospital nurses (Simmons et al., 2001). Following regulatory and payment reforms- most notably the 2000 Prospective Payment System, which shifted emphasis from clinical to administrative tasks (McCreary, 2020; Smith-Stoner, 2002; Tullai-McGuinness, 2008)- home health nurses began reporting some of the lowest job satisfaction levels among nursing specialties (Sochalski, 2004). More recently, the 2000 Patient Driven Groupings Model (PDGM) financially disincentivized and reduced therapy utilization (Medicare Payment Advisory Commission, 2024). Although the impact of the PDGM on the home health nursing workforce has not been measured, it is possible that reduced therapy services will increase care demand on nurses, shifting responsibilities that fall outside of their core training and skillsets. This is concerning given that unmanageable workloads have been linked to home health nurse dissatisfaction and turnover intention (Ellenbecker et al., 2006; McCreary, 2020; Narayan, 2023).

Given the growing demand for home health services coinciding with persistent concerns about nurse workforce instability, we sought to evaluate patterns of work transitions surrounding the home health sector relative to other care settings. Specifically, we aimed to (1) compare the likelihood of nurses leaving the home health versus the hospital sector—the largest employment sector for U.S. nurses (Smiley et al., 2023); (2) assess whether these differences evolved over time, particularly in the decades marked by increased home health nurse dissatisfaction; and (3) evaluate the likelihood that nurses from any other sector entered home health as new employment.

### New Contributions

A contribution of our analyses is that we focus on sector turnover. While past research has focused on organizational turnover (Anthony & Milone-Nuzzo, 2005; Canton et al., 2009; Smith-Stoner, 2004), sector turnover indicates switching the job setting or field in which a person is employed, such as a nurse leaving the hospital setting to work in home health care, or leaving health care altogether. Sector turnover, in contrast, may be due to factors such as chronic dissatisfaction or burnout, and is an important indicator of health care workforce capacity. Since sector turnover may reflect challenges that extend beyond individual organizations to health systems, we can begin to understand workforce losses, which pose the most significant implications for policy, resource allocation, and system capacity to meet care needs. In addition, this is the first paper to examine entry into the home health sector, offering a more complete picture of national home health workforce dynamics and the nation’s supply of home-based nursing services. Herein, we can better understand the workforce’s capacity to address large-scale care needs, particularly those of the growing population of older adults.

Predictors for modeling our outcomes of sector turnover and entry into home health are theoretically grounded in established economic theories of labor supply and labor market participation, which posit that individuals’ employment decisions are shaped by a combination of human capital characteristics (e.g., education, experience, and licensure), demographic and household factors (e.g., age, gender, marital status, and presence of young children), and job-specific attributes such as working hours or geographic labor market conditions (Becker, 1964; Mincer, 1974). These frameworks suggest that the same set of factors can influence both withdrawal from a sector and decisions to enter a new one, supporting our use of a unified covariate approach across outcomes. Applying a consistent predictor set allows us to disentangle whether common labor-market mechanisms—such as life-course constraints, opportunity costs, or sector-specific wage and working-condition differentials—may systematically shape nurses’ movement into and out of home health. This approach enhances comparability across models and yields clearer insights into the structural drivers of home health workforce dynamics.

## Methods

### Data and Sample

This was an observational study using data from the Current Population Survey (CPS), a nationally representative survey administered by the Census Bureau and Bureau of Labor Statistics, and the main source of information about the U.S. labor force. The CPS includes monthly data collected on a variety of employment-related characteristics for a wide range of industries and occupations. The CPS is administered via a multi-stage stratified probability sample of approximately 60,000 households in all 50 states of the United States and the District of Columbia. One reference person for each household typically responds on behalf of all members of the home, excluding individuals under age 15 or in the Armed Forces. Households are interviewed for 4 consecutive months, not surveyed for the next 8, then re-interviewed for 4 months. New groups are both introduced and rotated out of the CPS every month (U.S. Census Bureau, 2021). We pooled CPS monthly public use files from 2003 to 2023 from the IPUMS database, in which CPS data are compiled, published, and harmonized across time by the University of Minnesota (Flood et al., 2023; IPUMS-CPS, 2021).

In the CPS, a combination of occupation and industry codes allows identification of workers of specific occupations working within specific sectors. Industry classifications are based on the North American Industrial Classification System (NAICS) and include home health services as well as other health care and non–health care industries. Our sample included RNs and LPN/LVNs over age 18 responding to the CPS between January 2003 (when an industry code for home health was introduced) and June 2023. Following prior literature (Frogner & Dill, 2022), we harnessed the panel aspect of the CPS to observe individuals who responded to the survey multiple times. Data from the same individuals were linked over all available months of response to enable measurement of any instance of movement into or out of a sector. For our first two objectives, measuring movement out of home health and hospital sectors, we used a weighted sample of 1,562,781 survey responses from 307,932 unique RNs and LVN/LPNs who were employed in either home health or hospital sectors in at least one survey response. For our third objective, examining entry to home health, we expanded our sample to 2,378,737 responses from RNs and LVN/LPNs working in all possible sectors; this was done to capture all possible transitions into home health. We restricted our samples to respondents with nonmissing data on all key study variables.

### Measures

Outcomes: Our first outcome assessed monthly *sector turnover*, which was measured as departure from employment in the home health versus the hospital industry from one CPS observation period to the next. Because this involved measuring movement out of a sector in which an individual was employed in a previous month’s response, our outcome was measured by observing the respondents’ second survey observation. Sector turnover encompassed movement into either another sector or into unemployment. Our second outcome was *entry into the home health sector*, measured as employment in the home health sector following employment in another sector in a prior survey response. We included all transitions made by an individual in our outcomes, but did not track whether respondents moved out of one sector and later into another, or vice versa.

Exposure: For our objectives measuring transitions out of home health, our main independent variable was *home health versus hospital sector*, which was based on CPS industry codes. For our objectives measuring transitions into home health, we expanded this variable to include health care sectors outside of home health in which a nurse was previously employed; these included four *health care categories* (hospital, nursing home/residential care, ambulatory/provider offices, and “other” health care) along with one category which encompassed any non–health care sector of employment. Our definition and coding of health care sectors were based on the most commonly-identified CPS industry codes, where RNs and LPN/LVNs indicated employment and are detailed in the Appendix.

Covariates: Covariate measures included *licensure as RN versus LPN/LVN* based on the U.S. Census occupation classifications system. We also observed sociodemographic characteristics including *age over 18* and *age squared*, measured continuously; a binary *gender male/female indicator; race and ethnicity*, which was coded as Latine (whether the individual endorsed being of Hispanic, Latino or Spanish origin), and non-Latine Black, White, Asian/Pacific Islander, American Indian/Alaska Native, and other or multiple races. We acknowledge the complexity of measuring race and ethnicity in a survey context; efforts were made to allow as much detail and specificity as possible based on survey response, while balancing the need for sufficient observations in each category for analytical feasibility. Other sociodemographic characteristics included *presence of own child(ren) under 5 in the home* and *marital status* categorized as either married or in domestic partnership, widowed/divorced/separated, or never married. A variable for *highest education* was categorized as Diploma/Associate, Bachelor’s, Master’s/Doctorate, and other. A variable for *urbanicity* indicated whether a respondent’s household was located within or outside a metropolitan area, based on the Census Bureau’s delineation of a metropolitan statistical area as one with at least one urban area with 50,000 or more residents. One work-specific covariate measured whether the nurse was employed on a *part-time or full-time* basis; this variable was lagged so that it was linked to the work status in a sector job from which the respondent may or may not have turned over in a subsequent survey observation. A variable for *geography* divided the United States into nine regions based on the Census Bureau’s 1990 classifications. *Year dummies* controlled for variations in time between 2003 and 2023. Finally, to assess whether turnover patterns changed over time for home health nurses versus nurses working in hospitals, we included interaction terms of sector turnover and each year from 2003 to 2023.

We encountered constraints in the sample sizes of nurses entering home health when performing our analyses. Therefore, we collapsed and excluded certain covariates to maintain the statistical integrity of our models estimating home health sector entry. Specifically, time was collapsed into “pre-COVID” (2003–2019) and “post-COVID” years (2020–2023), race into a binary of White and non-White, and marital status into currently versus not currently married. Gender, children under 5, education, and region were excluded.

### Statistical Analyses

To compare home health with hospital RNs and LPN/LVNs, summary descriptive statistics were obtained using chi-square for categorical variables and bivariate ordinary least squares (OLS) regressions for continuous variables (Table 1). Descriptive statistics were measured once, at the first consecutive survey response for each of the 307,932 unique home health and hospital-based nurses.

**Table 1.** Descriptive Statistics of Nurses (RNs and LVN/LPNs) in the U.S. Workforce, January 2003 to June 2023.

Characteristic	Home health sector (7.99%)	Hospital sector (92.01%)	*p*
*Monthly sector turnover rate (%)*	4.85	2.12	<.001
*Nurse licensure (%)*			<.001
Registered nurse	72.2	91.1	
Licensed vocational/practical nurse	27.8	8.9	
*Age*	44.9	41.7	<.001
*Females (%)*	91.9	89.2	<.001
*Race and ethnicity (%)*			<.001
Latine	8.6	6.0	
White	66.4	71.6	
Black	18.1	11.6	
Asian Pacific Islander	4.6	9.1	
American Indian Alaska Native	0.7	0.4	
Other or multiple races	1.6	1.3	
*Children under 5 (%)*	12.5	15.3	<.01
*Marital status (%)*			<.001
Married/domestic partnership	56.1	61.8	
Widowed, divorced, separated	26.4	16.3	
Never married	17.5	21.9	
*Highest education (%)*			<.001
Diploma/Associate	42.2	29.0	
Bachelor of Science	33.0	53.3	
Masters/Doctorate	5.9	9.2	
Other	19.0	8.5	
*Urbanity (%)*			<.001
Metro/urban area	82.0	85.8	
Outside metro area	18.0	14.2	
*Work status (%)*			<.001
Full-time	74.6	78.8	
Part-time	25.4	21.2	
*Region (%)*			<.001
New England	5.8	5.3	
Middle Atlantic	14.2	13.5	
East North Central	14.8	16.4	
West North Central	6.7	8.1	
South Atlantic	21.5	19.6	
East South Central	5.8	6.9	
West South Central	17.0	11.2	
Mountain	4.9	5.5	
Pacific	9.4	13.6	

*Source.* Current Population Survey (CPS). χ^2^ and bivariate ordinary least squares regressions performed to test for systematic differences between home health and hospital nurses.

*Note.* Weighted *n* = 307,932 (unweighted *n* = 31,685). RN, registered nurse; LPN/LVN, licensed practical/vocational nurse. Descriptive characteristics were measured at respondents’ first consecutive observation in the CPS survey.

Next, we used logistic regression models to estimate sector turnover for home health compared with hospital nurses. This included 1,562,781 responses from home health and hospital-based nurses. We began with unadjusted models, then sequentially and additionally adjusted for sociodemographic, work status, and geography/time variables. We then assessed turnover over time using interaction terms of sector (home health and hospital) and year (each year from 2003 to 2023). For entry into the home health sector, we analyzed 2,378,737 observations from nurses working in other sectors, adjusting for covariates in a similar stepwise fashion. All regression results are reported using marginal effects and describe percentage point changes in the probability of an outcome, per unit change for continuous variables, and compared with the reference category for categorical variables. Specifically, our marginal effects describe the relative probability that RNs and LPN/LVNs *leave* the home health sector compared with the hospital sector, and that RNs and LPN/LVNs *enter* home health from a variety of other sectors (nursing home/residential care, ambulatory care, “other” health care, and non–health care sectors) compared with the hospital sector; as well as the contribution of other covariates in predicting turnover. We applied CPS survey weights to generalize results to the U.S. population. Our analyses were performed using Stata version 18.5.

Since our analytic approach involved pooling cross-sections of survey data linked by individuals’ responses over time, bias may exist from the nonindependence of responses. Although we used Stata’s “svy” complex survey suite of commands, which accounts for clustering (StataCorp, 2023), we conducted sensitivity analyses to assess potential bias from within-individual correlation. Specifically, we repeated all regressions using Stata’s vce(cluster) specification, which is an alternative variance estimator used to adjust standard errors in regression models for clustered data.

## Results

### Descriptive Statistics

Among 307,932 individual RNs and LPN/LVNs, 92% were employed in the hospital and 8% in the home health sector (Table 1). Hospital nurses were more likely to have more advanced RN licensure (91.1%) compared with home health nurses, who were 72.2% RNs and 27.8% LPN/LVNs. Nurses in the home health sector had lower educational preparation (42.2% diploma/associate and 33% bachelor-level degrees) compared with the hospital (29% diploma/associate and 53.3% bachelors). Home health sector nurses were older on average (mean age 44.9) than hospital nurses (mean age 41.7), somewhat less likely to have children under 5 (12.5% of home health versus 15.3% of hospital), and more likely to be widowed, divorced, or separated (26.4% of home health versus 16.3% of hospital). The majority of nurses in both sectors identified as White (66.4% home health and 71.6% hospital). Racial composition otherwise varied, with greater proportions of nurses in the home health sector identifying as Black (18.1%) compared with hospital sector nurses (11.6%), while fewer home health sector nurses identified as Asian or Pacific Islander (4.6%) compared with hospital sector nurses (9.1%). More nurses in the home health sector resided outside metropolitan areas (18%) than those working in the hospital sector (14.2%).

### Turnover Out of Home Health Versus Hospital Sectors

**Figure 1.**
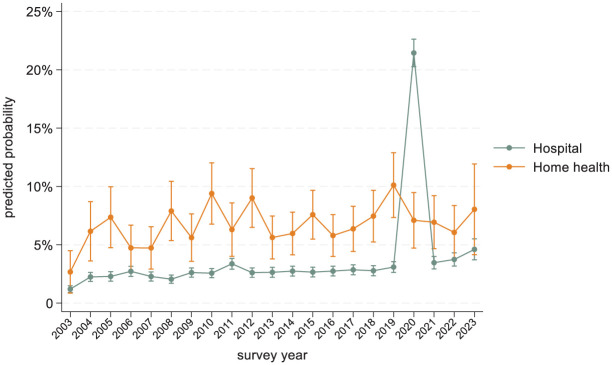
Predicted Probabilities of Monthly Turnover From Home Health and the Hospital Sector for RN and LPN/LVN Nurses From January 2003 to June 2023. *Note.* Weighted *n* = 2,378,737 (unweighted *n* = 248,535). Estimates are derived from logistic regression models with an interaction term of sector and year as the main regressor. Models were adjusted for nurse licensure (RN, registered nurse and LPN/LVN, licensed practical/vocational nurse), sociodemographic characteristics, and full-versus part-time work status.

Marginal effects from regression models estimating sector turnover from home health compared with the hospital sector are reported in Table 2. Home health nurses were significantly more likely to report sector turnover than hospital nurses. In unadjusted analyses, the predicted probability of nurse turnover from the home health sector was higher (7.2%) compared with the hospital sector (3.7%), yielding a significant 3.5 percentage points (pp) difference (95% CI 3.0–4.1 pp). This difference fell to 2.9 pp (95% CI 2.3–3.4 pp) after adjusting for all covariates. Across both groups of nurses, having more advanced RN licensure modestly but significantly reduced likelihood of sector turnover by about 1 pp. Adjusted predicted probabilities with the interaction of sector and year are shown in Figure 1, depicting turnover from 2003 to 2023. Through the years, turnover from the home health sector was several percentage points higher, with predicted probabilities of turnover hovering between about 5% and 10%, and subject to greater year-to-year variation than turnover from the hospital sector. The sole exception to this trend was in 2020, when turnover from the hospital sector surged while turnover from home health followed its established pattern. By 2021, hospital sector turnover declined to near pre-pandemic levels and was once again surpassed by turnover from the home health sector in 2021.

**Table 2.** Marginal Probabilities of Monthly Turnover Among RNs and LPN/LVNs for Home Health Versus Hospital Sector and Other Nurse Characteristics.

Marginal effect (95% CI)								
	Model 1. Unadjusted	*p*-value	Model 2. Socio-demographic	*p*-value	Model 3. Work status	*p*-value	Model 4. Geography and time^a^	*p*-value
*Sector*								
Hospital [ref]								
Home health	3.5 (3.0, 4.1)	<.001	3.2 (2.7, 3.8)	<.001	3.2 (2.6, 3.7)	<.001	2.9 (2.3, 3.4)	<.001
*Nurse licensure*								
Licensed vocational/practical nurse [ref]								
Registered nurse			−1.1 (−1.7, −0.6)	<.001	−1.1 (−1.7, −0.6)	<.001	−0.9 (−1.4, −0.4)	<.001
*Age*			0.0 (−0.1, 0.0)	.387	0.0 (−0.1, 0.0)	.410	0.0 (0.0, 0.1)	.278
*Age* ^2^			0.0 (0.0, 0.0)	.128	0.0 (0.0, 0.0)	.146	0.0 (0.0, 0.0)	.595
*Gender binary*								
Male [ref]								
Female			−0.1 (−0.5, 0.3)	.686	−0.1 (−0.5, 0.3)	.587	0.1 (−0.2, 0.5)	.476
*Race and ethnicity*								
Latine [ref]								
White			−0.6 (−1.2, −0.1)	.022	0.7 (−1.2, −0.1)	.018	0.1 (−0.4, 0.6)	.725
Black			0.2 (−0.4, 0.1)	.460	0.3 (−0.4, 0.9)	.443	0.8 (0.2, 1.4)	.013
Asian Pacific Islander			−0.6 (−0.4, 0.9)	.065	−0.6 (−1.3, 0.0)	.067	0.1 (−0.6, 0.6)	.893
American Indian Alaska Native			0.7 (−1.3, 2.7)	.476	0.7 (−1.3, 2.8)	.474	1.1 (−0.7, 3.0)	.213
Other or multiple races			−0.7 (−2.0, 0.5)	.264	−0.8 (−2.0, 0.5)	.251	−0.4 (−1.6, 0.7)	.443
*Children under 5*			0.6 (0.2, 1.0)	.005	0.5 (0.1, 0.9)	.009	0.5 (0.1, 0.8)	.014
*Marital status*								
Married/domestic partnership [ref]								
Widowed, divorced, separated			0.0 (−0.3, 0.3)	.975	0.0 (−0.3, 0.3)	.875	0.1 (−0.2, 0.4)	.471
Never married			0.4 (0.0, 0.7)	.058	0.4 (0.0, 0.7)	.048	0.2 (−0.2, 0.5)	.357
*Highest education*								
Diploma/Associate [ref]								
Bachelor of Science in Nursing			0.2 (−0.1, 0.5)	.143	0.2 (−0.1, 0.5)	.141	−0.2 (−0.4, 0.1)	.219
Masters/Doctorate			0.5 (0.0, 0.9)	.055	0.5 (0.0, 0.9)	.049	0.0 (−0.4, 0.4)	.957
Other			−0.5 (−0.9, 0.0)	.029	−0.5 (−0.9, −0.1)	.028	−0.3 (−0.8, 0.2)	.209
*Urbanity*								
Outside metro area [ref]								
Metro area			0.3 (0.0, 0.6)	.030	0.3 (0.0, 0.6)	.035	0.1 (−0.2, 0.4)	.639
*Work status*								
Part-time [ref]								
Full-time					−0.3 (−0.6, 0.0)	.046	−0.4 (−0.7, −0.1)	.003
*Region*								
New England [ref]								
Middle Atlantic							0.4 (−0.1, 0.9)	.113
East North Central							−0.3 (−0.7, 0.2)	.289
West North Central							0.0 (−0.5, 0.5)	.956
South Atlantic							0.5 (0.0, 0.9)	.059
East South Central							−0.1 (−0.6, 0.5)	.791
West South Central							0.2 (−0.3, 0.8)	.358
Mountain							0.6 (0.1, 1.2)	.030
Pacific							0.8 (0.3, 1.3)	.002

*Source.* Current Population Survey, January 2003 to June 2023.

*Note.* Weighted *n* = 1,562,781 (unweighted *n* = 161,345). RN, registered nurse; LPN/LVN, licensed practical/vocational nurse. Marginal effects are multiplied by 100 and report the percentage point (pp) change in the predicted probability of sector turnover per unit change of covariate or compared with the reference category, controlling for all other covariates in the model.

a Model 4 is also adjusted for the years 2003–2023.

### Entry Into the Home Health Sector

Entry into home health from other sectors occurred infrequently and at comparable rates across sectors. In comparing relative likelihoods of entering home health, we found that nurses were modestly more likely to do so after leaving nonhospital sectors than after leaving the hospital sector (Table 3). The largest differences were identified in the “other” health care category, from which there was 1.0 pp (95% CI 0.8–1.2) increased likelihood of transitioning into home health employment, and in the nursing home/residential care sector, from which there was a 0.5 pp (95% CI 0.4–0.7) increased likelihood of transitioning into home health employment (both *p* < .001). The overall probability of transitioning into home health was low across sectors, ranging from 0.17% following previous employment in the hospital sector, 0.7% following previous employment in the nursing home/residential care sector, and 1.2% following previous employment in the “other” health care sector category (Table 4).

**Table 3.** Marginal Probabilities of Entering Employment Into Home Health From Another Employment Sector Among RNs and LVN/LPNs.

Marginal effect (95% CI)								
	Model 1. Unadjusted	*p*-value	Model 2. Socio-demographic	*p*-value	Model 3. Work status	*p*-value	Model 4. Geography and time	*p*-value
*Nurse sector*								
Hospital [ref]								
Nursing home/residential	0.5 (0.4, 0.7)	<.001	0.5 (0.4, 0.6)	<.001	0.5 (0.4, 0.6)	<.001	0.5 (0.4, 0.7)	<.001
Ambulatory	0.1 (0.0, 0.2)	.002	0.1 (0.0, 0.2)	.002	0.1 (0.0, 0.2)	.002	0.1 (0.0, 0.2)	.002
Other health care	1.0 (0.8, 1.2)	<.001	1.0 (0.8, 1.2)	<.001	1.0 (0.8, 1.2)	<.001	1.0 (0.8, 1.2)	<.001
Other non–health care	0.4 (0.3, 0.5)	<.001	0.4 (0.3, 0.5)	<.001	0.4 (0.3, 0.5)	<.001	0.4 (0.3, 0.5)	<.001
*Nurse licensure*								
Licensed vocational/practical nurse [ref]								
Registered nurse			0.0 (−0.1, 0.1)	0.945	0.0 (−0.1, 0.1)	0.951	0.0 (−0.1, 0.1)	.836
*Age*			0.0 (0.0, 0.0)	0.414	0.0 (0.0, 0.0)	0.396	0.0 (0.0, 0.0)	.267
*Age* ^2^			0.0 (0.0, 0.0)	.487	0.0 (0.0, 0.0)	.464	0.0 (0.0, 0.0)	.318
*Race binary*								
White [ref]								
Non-White			0.0 (0.0, 0.1)	.632	0.0 (0.0, 0.1)	.599	0.0 (−0.1, 0.1)	.769
*Marital status*								
Not married [ref]								
Married/domestic partnership			−0.1 (−0.1, 0.0)	.007	−0.1 (−0.1, 0.0)	.006	−0.1 (−0.1, 0.0)	.007
*Urbanity*								
Outside metro area [ref]								
Metro area			0.0 (−0.1, 0.1)	.977	0.0 (−0.1, 0.1)	.988	0.0 (−0.1, 0.1)	.934
*Work status*								
Part-time [ref]								
Full-time					0.0 (−0.1, 0.0)	.575	0.0 (−0.1, 0.0)	.472
*Time period*								
Pre-COVID [ref]								
Post-COVID							0.2 (0.1, 0.3)	.000

*Source.* Current Population Survey, January 2003 to June 2023.

*Note.* Weighted *n* = 2,378,737 (unweighted *n* = 248,535). Marginal effects are multiplied by 100 and report the percentage point (pp) change in the predicted probability of entering the home health sector per unit change of covariate or compared with the reference category, controlling for all other covariates in the model.

**Table 4.** Predicted Probabilities of Entering Home Health From Another Sector.

Sector	%
Hospital	0.17
Nursing home/residential	0.7
Ambulatory	0.3
Other health care	1.2
Other non–health care	0.6

*Source.* Current Population Survey, January 2003 to June 2023.

*Note.* Weighted *n* = 2,378,737 (unweighted *n* = 248,535). Predicted probabilities were retrieved from logistic regression models estimating entry into the home health sector, where employment in a non-home health sector in an earlier survey response was the main predictor. Models were adjusted for nurse licensure (RN, registered nurse, and LPV/LVN, licensed practical/vocational nurse), sociodemographic characteristics, and a binary time period of 2003 to 2019 and 2020 to 2023 to represent years before and after the onset of the COVID-19 pandemic.

In our sensitivity analyses, regression models validated with Stata’s vce(cluster) option produced virtually identical results to our reported results produced using the “svy” command, with odds ratio coefficient magnitudes varying at most by tenths of points. This suggests robustness in our models against bias from correlation among responses within individuals.

## Discussion

In this study of sector transitions, we found that nurses were consistently more likely to depart the home health sector than the hospital sector over a 20-year timeframe, except during an isolated surge of hospital-based nurse turnover coinciding with the onset of the COVID-19 pandemic. We also found that the probability of nurses entering home health from other fields was quite low, with predicted probabilities of home health sector entry under or around 1% from any other sector. Addressing these national, sector-level patterns requires consideration of systemic challenges and opportunities across the home health workforce and industry.

Our findings on low entry coupled with higher departure align with research that show declines in both the sheer number and share of U.S. nurses endorsing home health as a practice setting over the last decade (Smiley et al., 2023). Although it is difficult to confirm the total accuracy of our very low home health entry estimates, our overall findings are complemented by data showing high home health nurse job turnover and vacancy rates (Hospital and Healthcare Compensation Service, 2022). This is concerning, given the growing demand for home health services, and bodes poorly for the ability of RNs and LPN/LVNs to play roles in addressing growing home health needs.

While no demographic traits strongly predicted turnover in our data, the demographic differences that we noted between home health and hospital-based nurses reveal urgent challenges and opportunities for strengthening the home health nursing workforce. For instance, consistent with prior research, home health nurses in our sample were older on average (Jarrín et al., 2017; Smiley et al., 2023), suggesting longer career tenure- likely due to traditional hiring practices that favor experienced clinicians (Patterson et al., 2013). To the extent that age reflects tenure, we found no evidence that it was protective against turnover. This contrasts with research that has linked age and tenure to retention among home health nurses (Ellenbecker et al., 2008; Tourangeau et al., 2017). In addition, concerns about accelerated losses of experienced nurses from the U.S. workforce (Smiley et al., 2023) are particularly acute in the home health sector, given its older workforce. Our findings, therefore, underscore an urgent need to attract newer nurses to home health.

Among factors associated with turnover, the presence of a child under 5 emerged as a modest yet potentially meaningful consideration. This finding is particularly salient given our results suggesting an older and contracting workforce, as well as the observation that nurses with children under 5 were less prevalent in home health compared with the hospital sector. In the United States, where child care for young children is neither universally available nor cost-free, scheduling may be a significant concern. Flexible scheduling may be more feasible in home health than in hospital or office-based roles with rigid hours, and thus potentially appealing to nurses with young families. Unfortunately, challenges related to scheduling are well-documented in the home health literature, where erratic and long, unpredictable hours have been linked to burnout, turnover, adverse mental health, and moral distress by encroaching on family and personal life and reducing time with patients (Anthony & Milone-Nuzzo, 2005; Bergman et al., 2021; Ikeda et al., 2021; Petersen & Melzer, 2023). Conversely, balanced lifestyle (Jarrín et al., 2017) and flexible scheduling have been linked to work-life balance, patient care continuity and intent-to-stay in home health nursing jobs (Anthony & Milone-Nuzzo, 2005; Garza & Taliaferro, 2021; Tourangeau et al., 2014, 2017). Identifying strategies to systematically improve scheduling and workload management may therefore be instrumental in attracting and retaining a new demographic of nurses to the home health sector.

Attracting and retaining newer-career home health nurses may also warrant reconsideration of conventional hiring practices, including educational requirements. In our sample, significantly larger proportions of home health nurses held less advanced degrees (diploma and associate vs. bachelors) and licensure (LPN/LVN vs. RN) than hospital nurses. Prompted by national bodies (Institute of Medicine, 2011) the nursing workforce overall has gravitated toward earning more advanced bachelor’s over diploma or associate’s degrees (Smiley et al., 2023). While this trend has advanced professionalism within the nursing field overall, maintaining educational thresholds may create barriers to entry for prospective home health nurses, especially since this sector has traditionally drawn from a more diverse mix of educational backgrounds. Although RN-driven care may be appropriate in higher-acuity hospital settings (Lasater et al., 2024), evidence suggests that LPN/LVNs can safely play critical and complementary roles to RNs in home health; for instance, alternating LVN/LPN visits with RN visits for patients requiring technical skills (e.g., wound care) can improve patient outcomes and free up time for RNs to perform more complex and supervisory tasks, such as case management, care plan creation and provision of continuous care for sicker and complex patients (D’Errico & Lewis, 2010). Educational discrepancies could also be addressed via specialized training programs, which have been shown at the workplace level to support job retention in early career and newly hired nurses (Linscheid & Bell, 2021; Pennington & Driscoll, 2019) as well as skill development and retention, similarly for associate and bachelor-prepared home health nurses (Rosenfeld et al., 2010). Precedent also exists for state-sponsored workforce development programming for home care aides (Bryant et al., 2021) and trade association-directed post-graduate nurse residency programs focused on long-term care (Oregon Health Care Association, n.d.). Similar models could be adapted to strengthen a home health nursing pipeline without broadly elevating education or training requirements.

Beyond training, additional measures to support entry of new nurses into home health could include strengthening access to clinical support, which is often limited in the field and may be unattractive or potentially unsafe for newer nurses unprepared for high degrees of clinical autonomy. Medicare-certified home health agencies are not required to employ prescribing providers, like physicians, nurse practitioners, or physician assistants (U.S. Government Publishing Office, 42 C.F.R. § 424.22, 2025). Although initial and updated home health orders must be written by community or hospital-based providers, their continued involvement in care coordination and delivery is limited (Boyd et al., 2018), and timely access to providers for guidance may be challenging. Enhancing clinical supports, via dedicated providers and harnessing telehealth capabilities, could enable newer nurses to practice more safely and confidently in clients’ homes.

Although elevated turnover out of the home health sector is concerning for workforce capacity, job transitions can also reflect desirable tendencies, such as career advancement or movement of workers for whom home health practice was a poor fit. The sector may benefit from providing opportunities for career advancement, such as formal clinical ladder programs commonplace in hospital nursing, or more diverse roles for nurses.

Our findings, derived from a large, nationally representative sample of nurses and not exclusive to specific workplaces or geographic locales, may provide insights for broad, system-level reform. The Centers for Medicare and Medicaid Services (CMS), as the body that regulates, finances, and monitors the quality of the Medicare home health care benefit for older Americans, could play a role. Similar to calls for CMS to exercise influence via Conditions of Participation for safe nurse staffing in hospitals (Aiken & Fagin, 2022), influence could be exerted on home health agencies, with labor- and staffing-specific benchmarks tailored to home health workplace concerns. Links between nurse working conditions and better patient care and nurse retention in home health settings (Jarrín et al., 2014, 2017) suggest that CMS could advance quality goals, save costs, and support nurse staffing by addressing workplace concerns highlighted in the literature. For instance, schedule volatility (Bergman et al., 2021), unrealistic productivity requirements, excessive workloads, long hours, and uncompensated time (Ellenbecker et al., 2006; Giltenane et al., 2022; Tullai-McGuinness, 2008) could be addressed via requirements around limits on daily patient visit volumes and guaranteed pay provisions.

Other analyses of the RN workforce suggest recent shifts away from hospital employment toward ambulatory and community-based settings (Auerbach et al., 2024), possibly reflecting a growing interest in community-oriented care. Our findings on cross-sector entry to home health, although modest in magnitude, similarly indicate a greater inflow of nurses into home health from nonhospital settings (e.g., ambulatory and nursing home settings) and may reflect overlapping interests in nonhospital care environments or patient populations. Reenvisioning the roles of RNs and LPN/LVNs within the spectrum of home health care services in the United States could build on these trends, creating more appealing career opportunities while expanding workforce capacity to meet the needs of the aging population. Most home health RNs and LPN/LVNs currently work under the episodic Medicare home health benefit. Home-based primary care (HBPC), which is functionally separate from Medicare home health, provides comprehensive, long-term services shown to improve outcomes at reduced costs (De Jonge et al., 2014; Ornstein et al., 2021; Sun et al., 2022). However, HBPC’s growth is constrained by a limited workforce comprised of physicians and nurse practitioner “providers” who face time pressures from travel and the complex needs of homebound patients (Yao et al., 2021). Integrating RNs and LPN/LVNs into HBPC teams could be a cost-effective way to expand reach and efficiency. The CMS *Making Care Primary* pilot (Centers for Medicare and Medicaid Services, 2024) aims to strengthen primary care through community-based supports, which aligns with calls to expand and formalize RNs’ roles in primary care to promote professional satisfaction by maximizing RN skillsets (Nikpour et al., 2023) and support workloads of overburdened primary care providers (Porter et al., 2023). Leveraging home health RNs’ skills in HBPC could involve care coordination and case management, alternating visits with providers, or performing hybrid in-person telehealth visits in which providers join remotely.

### Limitations

Our analysis had several limitations. As an observational study, our findings are limited to associations and lack insight on causes of turnover. Our dataset also lacked information on key work-related characteristics, such as earnings, scheduling, and union membership; although the CPS Annual Social and Economic Supplement (ASEC) contains this information, smaller ASEC sample sizes of home health nurses made its use impossible for this analysis. In addition, our measure of turnover did not differentiate between transitions into unemployment and those into other sectors, as the number of nurses who became unemployed was too small for meaningful analysis. Entry to home health was also measured with a more restricted set of covariates because of the smaller samples endorsing entry, which limits the interpretation of the relationship between nurse characteristics and home health entry. For instance, we were unable to measure the effect of young children on job movement into home health, which may theoretically play a larger role than characteristics that we were able to measure, such as marital status. Finally, it is important to note that the CPS has been shown to underestimate sectoral job movement relative to administrative records (Hyatt & Spletzer, 2013). While the consistency of our results over time supports our conclusions, we issue caution against overinterpreting exact numbers and encourage studies to use alternative data sources to validate these patterns.

## Conclusions

This study provided new insights into the movement of RNs and LPN/LVNs in and out of the home health sector using a nationally representative sample over the last two decades. We identified consistent patterns of nurses leaving the home health sector and few nurses from other sectors moving into home health. Relative to other specialties, home health may be prone to more accelerated losses of nursing talent due to factors like the older age of its workforce and educational and experiential barriers to practice. Our findings highlight the urgent need to attract and retain nurses in the home health sector, which may demand broad policy reforms and reimagining the roles of RNs and LPN/LVNs within the home health landscape. Home health may be an attractive sector for nurses who value autonomy, flexibility, and community-based work. We encourage research that explores strategies to enhance these positive features of home health nursing while addressing potential deterrents.

## Appendix

**Appendix:** Census Bureau Occupation and Industry Codes Used to Define Nursing Occupation and Health Care Sectors in Analytic Sample

Nursing occupation	Census Bureau occupation code and label	
Registered Nurse	3,255	Registered nurses
	3,130	Registered nurses
Licensed Practical/Vocational Nurse	3,500	Licensed practical and licensed vocational nurses
Health care sector	Census Bureau industry code and label	
Home health	8,170	Home health services
Hospital	8,190	Hospitals
	8,191	General medical and surgical hospitals, and specialty (except psychiatric and substance abuse) hospitals
	8,192	Psychiatric and substance abuse hospitals
Nursing home/long-term care	8,270	Nursing care facilities (skilled nursing facilities)
	8,290	Residential care facilities, except skilled nursing facilities
Outpatient/ambulatory	7,970	Offices of physicians
	8,070	Offices of optometrists
	8,080	Offices of other health practitioners
	8,090	Outpatient care centers
Other health care	8,180	Other health care services

*Source.* IPUMS CPS.

*Note.* Industry codes from 2003–2010, 2011–2019, 2020+ occupation codes to 2003–2008, 2009–2013, 2014–2019, and 2020+ industry codes.
